# Evaluating the implementation related challenges of *Shasthyo Suroksha Karmasuchi* (health protection scheme) of the government of Bangladesh: a study protocol

**DOI:** 10.1186/s12913-018-3337-x

**Published:** 2018-07-16

**Authors:** Sayem Ahmed, Md. Zahid Hasan, Mohammad Wahid Ahmed, Farzana Dorin, Marufa Sultana, Ziaul Islam, Andrew J. Mirelman, Clas Rehnberg, Jahangir A. M. Khan, Mahbub Elahi Chowdhury

**Affiliations:** 1 Universal Health Coverage Programme, Health Systems and Population Studies Division, icddr,b, 68 Shahid Tajuddin Ahmed Sharani, Mohakhali, Dhaka, 1212 Bangladesh; 2 Health Economics and Policy Research Group, Department of Learning, Informatics, Management and Ethics (LIME), Karolinska Institutet, Stockholm, Sweden; 3 Nutrition and Clinical Services Division, International Centre for Diarrhoeal Disease Research, Dhaka, Bangladesh; 4 School of Health and Social Development, Deakin University, Melbourne, Australia; 5 Centre for Health Economics, University of York, York, UK; 6 Department of Clinical Sciences, Liverpool School of Tropical Medicine, Liverpool, UK

**Keywords:** *Shasthyo Surokhsha Karmasuchi* (SSK), Health protection scheme, Implementation challenges, Implementation research, Process documentation, Research protocol, Bangladesh

## Abstract

**Background:**

Rapidly increasing healthcare costs and the growing burden of non-communicable diseases have increased the out-of-pocket (OOP) spending (63.3% of total health expenditure) in Bangladesh. This increasing OOP spending for healthcare has catastrophic economic impact on households. To reduce this burden, the Health Economics Unit (HEU) of the Ministry of Health and Family Welfare has developed the *Shasthyo Surokhsha Karmasuchi* (SSK) health protection scheme for the below-poverty line (BPL) population. The key actors in the scheme are HEU, contracted scheme operator and hospital. Under this scheme, each enrolled household is provided 50,000 BDT (620 USD) coverage per year for healthcare services against a government financed premium of 1000 BDT (12 USD). This initiative faces some challenges e.g., delays in scheme activities, registering the targeted population, low utilization of services, lack of motivation of the providers, and management related difficulties. It is also important to estimate the financial requirement for nationwide scale-up of this project. We aim to identify these implementation-related challenges and provide feedback to the project personnel.

**Methods:**

This is a concurrent process documentation using mixed-method approaches. It will be conducted in the rural Kalihati Upazila where the SSK is being implemented. To validate the BPL population selection process, we will estimate the positive predictive value. A community survey will be conducted to assess the knowledge of the card holders about SSK services. From the SSK information management system, numbers of different services utilized by the card holders will be retrieved. Key-informant interviews with personnel from three key actors will be conducted to understand the barriers in the implementation of the project as per plan and gather their suggestions. To estimate the project costs, all inputs to be used will be identified, quantified and valued. The nationwide scale-up cost of the project will be estimated by applying economic modeling.

**Discussion:**

SSK is the first ever government initiated health protection scheme in Bangladesh. The study findings will enable decision makers to gain a better understanding of the key challenges in implementation of such scheme and provide feedback towards the successful implementation of the program.

**Electronic supplementary material:**

The online version of this article (10.1186/s12913-018-3337-x) contains supplementary material, which is available to authorized users.

## Background

Rapidly increasing healthcare cost and the growing burden of non-communicable diseases have increased the out-of-pocket (OOP) spending (63.3% of total health expenditure) in Bangladesh [1]. This increasing OOP spending for healthcare has catastrophic economic impact on households, especially on the poor [2–4]. The National Health Policy of 2011 acknowledged that health is a human right and to achieve universal health coverage, it is necessary to ensure health services for the poor at an affordable cost [5]. For achieving this, the high burden of OOP payment must be decreased and financial protection for healthcare should be ensured. The Government of Bangladesh adopted the Health Care Financing Strategy 2012–2032 with a view to bringing all the citizens under the financial protection for healthcare by 2032 [6]. To achieve this goal, the Health Economics Unit (HEU), a wing of the Ministry of Health and Family Welfare (MoHFW) of the Government of Bangladesh has developed Shasthyo Surokhsha Karmasuchi (SSK), a health protection scheme [6]. Although the SSK has a comprehensive plan to cover all population, initially it is implementing targeting the below poverty line (BPL) population only.

### Shasthyo Surokhsha Karmasuchi (SSK)

The HEU of the MoHFW has developed the social health protection scheme (SSK) with the support from German Development Cooperation through KfW (German Development Bank) and GFA Consulting Group. Adopting the mechanism of health insurance model, the scheme was developed over a three-year period of extensive consultations with the experts. Currently, the scheme is being implemented at rural Kalihati Upazila (sub-district). The key actors in the scheme are HEU, contracted scheme operator, and Kalihati Upazila Health Complex (UpHC) [7].

#### The SSK cell

The SSK Cell (a group of personnel) has been formed by the HEU to work as the key management body for implementing the SSK project. The SSK Cell performs like an insurance providing organization. It formulates policy decisions and responsible for implementing the scheme activities through engaging hospitals and a Scheme Operator (SO). The SSK Cell performs administrative tasks, namely, project co-ordination, finance management, target population management, benefit package management, grievance process, and monitoring and evaluation.

#### Scheme operator (SO)

The SSK Cell contracted an insurance agency for providing SSK service management support to them at the UpHC and Tangail District Hospital (DH). Currently, the Green Delta insurance company has been contracted as SO. The SO is responsible for visiting the BPL households (enlisted based on selection criterion) to provide health card. They also facilitate the UpHC in claim reimbursement process, assist card holders in receiving healthcare services from UpHC and DH, and monitor the scheme activities.

#### SSK benefit package

Under the scheme, SSK members receive only inpatient healthcare at the UpHC and structured referral care from the DH. An electronic health card is provided to each enrolled household ensuring 50,000 BDT per year equivalent healthcare service coverage for 70 different disease groups (Table 1). The premium for this coverage is 1000 BDT per year that is financed by the government. Membership in SSK has many advantages compared to the regular patients in the public healthcare facility: free consultation for outpatient care, free inpatient care, free referral care from DH, access to a grievance authority for complaining on the quality of the services, and free access to essential drugs at UpHC and DH for inpatient care.

**Table 1:** SSK benefit package

Premium	Health services				Coverage
1000 BDT per household per year	Inpatient care: Inpatient care for 70 different diseases	Hospital bed and food: Provide hospital bed and food free of cost	Structured referral: Transportation cost for referral	Medicine and diagnostics: Free drugs and diagnostic	50,000 BDT per household per year

#### Claim management process

The hospitals (UpHC and DH) are reimbursed by SSK Cell within 30 days for providing free healthcare services to the SSK members based on verifiable patient records (claims). Reimbursement follows a case and diagnosis based payment systems using a simplified Diagnosis Related Groups (DRG) on 70 diseases. The hospitals submit the claim documents to the SO. The SO checks and sends these claim documents to the SSK Cell. The SSK Cell verifies the claims and invoice to the SO. Finally, the SO makes payment to the hospitals. With the extra funds the UpHC have fiscal space to expand the service list and improve the quality, so they can meet the quality criteria.

#### Information management system

The SSK Cell maintains a data warehouse with the help of Management Information System (MIS) of Directorate General of Health Services (DGHS). The SSK data server is hosted at DGHS-MIS center with free of charge and they provide general and maintenance support services. The hospital is equipped with a computerized hospital management system initially focusing on the member management and inpatient management. The system is based on customized software that includes patient registration, diagnosis, treatment, referral, discharge, and automated reporting which are useful for claim management and fraud control. The SO managed SSK booths at the hospitals maintain the membership related information. These booths are responsible for checking the membership status of SSK card holders before seeking any treatment from the hospital.

Through informal discussion and anecdotal evidences, many implementation-related challenges of the SSK project have been identified. These include delays in carrying out the assigned activities, failure to register target population as per selection criteria, low level of utilization of services by the SSK card holders, lack of motivation of the providers in dealing with the additional workload, management and administrative difficulties in smoothly operating all the activities for the SSK project. Therefore, there is a need to systematically document these implementation-related challenges of the SSK project and provide timely feedback to the project personnel for necessary refinement in the implementation.

### General objectives and research questions

The overall objective of this study is to identify the implementation related challenges of the SSK project and provide timely feedback to the project personnel for necessary refinement. The specific objectives of this implementation research are:

1. To review and validate the selection process of the BPL population for the SSK
2. To assess knowledge of SSK BPL card holders about the benefit package of the SSK
3. To document the barriers in utilization of the SSK services by the card holders
4. To record the service utilization pattern at the health facilities by the SSK card holders
5. To document the implementation related challenges of the SSK project and gather possible suggestions for addressing those challenges
6. To estimate the costs of scaling-up the SSK project nationwide

## Methods

### Study setting

The study will be conducted in the Kalihati Upazila under Tangail district where the SSK is currently being implemented. A total of 89,351 households (including 35,740 BPL households) of the Upazila will be the study population. The Kalihati Upazila Health Complex, the first contact point of the SSK beneficiaries, and Tangail District Hospital, the referral facility, will also be within the jurisdiction of this study.

### Design & Methods

This study will be a concurrent process documentation using mixed-method approach that includes both quantitative and qualitative assessments. The integrated approaches will provide the flexibility to fill in gaps in the available information, strengthen the validity of the assessment and provide different perspectives on contextual and multi-dimensional phenomena. The study will have 6 different phases. The different research activities planned to be implemented at different phases are shown in Table 2.

**Table 2:** Study activities

Activities	P-I* (1-3 m**)	P-II (4-6 m)	P-III (7-9 m)	P-IV (10-12 m)	P-V (13-15 m)	P-VI (16-18 m)	Data sources
Study protocol development and research review and ethical review committee approval	√						Not applicable
Review and validate the selection process of BPL population		√					Survey of member and non-member households
To assess knowledge of BPL card holders and document the barriers in utilization of the SSK services			√				Separate survey of member household (community survey) and focus group discussions (FGDs)
Review of service statistics at the health facilities to assess service utilization pattern among the card holders			√	√	√		Facility record review
Process documentation to assess progress in project implementation and identify related barriers			√	√	√		Document review and synthesis of secondary data
Key-informant interviews of the providers, managers, scheme operators to document implementation challenges and solutions		√		√			Key-informant interviews
Cost-analysis				√			Interviews with the SSK project and the hospital management personnel
Periodic feedback and follow up of the progress			√	√	√		Findings from the research activities
Reporting and dissemination						√	Findings from the research activities

^a^*P=*Phase*,* ^b^*m =* month

### Review and validate the selection process of BPL population for SSK

To understand the pitfall in existing BPL population identification we will review the method applied and tools used in this process. In addition, the problems in applying the selection criteria will be recorded through process documentation and key-informant interviews of the program personnel. Using appropriate quantitative approach targeted beneficiaries’ perspectives will also be collected to record the challenges in selection of the BPL population.

### Validation study

To validate the selection process of BPL population, we will estimate positive predictive value. Both SSK member and non-member households will be interviewed. For member household, a sampling frame will be collected from SSK project and from that frame the required number of samples will be selected randomly. For non-member, closest adjacent household of SSK member will be selected. If the closest adjacent household is found a member household of SSK project the next closest will be selected for interview. The heads of the selected households will be interviewed with a structured questionnaire on household characteristics, BPL selection criteria of the SSK, and detailed consumption expenditure information [see Additional file 1]. To identify the poor household, the average monthly consumption expenditure of each household will be compared with the poverty line defined by Bangladesh Bureau of Statistics (BBS) for Dhaka Division using cost-of-basic needs (CBN) approach [8]. This poverty line will be used as a gold standard for poverty identification in this study.

### Community survey

The community survey will be conducted to assess the knowledge of the card holders about SSK services as well as to document the barriers in utilization of such services. From the sampling frame of the SSK card holders, the respondents will be randomly selected. In this survey, the card holders will be asked whether they know about the benefit package of the SSK. They will also be asked whether they face any difficulties while receiving SSK services such as negligence of provider, unavailability of listed services, shortage of prescribed medicines, long waiting time, and unofficial tips. An instrument for assessing knowledge level is developed to gather this information which will be piloted before finalization. Focus Group Discussions (FGD) will be applied for understanding the experience, perception of beneficiaries about the SSK services, and barrier to utilize these services. Beneficiaries who utilized healthcare in last 3 months will be included in FGDs. FGDs will be held in an independent place away from the health facility. In each FGD, 8–10 participants from same level will participate. Initially, a number of 5 FGDs is planned. If the research team feels that additional knowledge can be extracted from more FGDs, then additional sessions will be organized.

### Facility record review for service utilization

From computer based record managed by the SSK project, numbers of different services utilized by the card holders will be retrieved. Facility record review will be done in 3 phases. In each phase, last 3 months patient's records will be gathered. Trend analysis will be done. Number of patients treated by disease, types of diagnostic services offered, type of drugs provided, and number of patient referred by disease along with compliance will be estimated.

### Key-informant interviews (KIIs)

The rationale of choosing key-informant interviews (KIIs) for this study is to understand the systems that affect barriers in implementation of SSK project activities as per plan and gather their suggestions. This will include delay in project implementation, problem in selection process of BPL population, availability of necessary equipments, drugs, logistics for providing services, scarcity of manpower, workload related issues, problem in referrals, problem related to SSK fund management, and barriers in claim management.

KIIs will be conducted face-to-face by experienced qualitative researchers. The interviewer would schedule a convenient time and place for the interview. The interview will be digitally recorded after having permission from the key-informant personnel. Another researcher will also take simultaneous verbatim notes. The duration of a KII will be at least 45 minutes to one-hour.

### Process documentation

The process documentation will be undertaken to review the progress in SSK project implementation activities, identify barriers for possible delays in implementation, scheme operator’s oversight, and how well the outputs of the SSK project are aligned to achieve outcomes and impacts. The areas of process documentation include number of services under benefit package, enrollment of the beneficiaries, service provision steps, claim management and payment process to the provider. Multiple methods will be used for capturing information in process documentation (e.g. document review and synthesis of secondary data). Through process documentation timely feedback will be provided to the SSK project personnel.

### Cost analysis

The additional cost of scaling-up the SSK project at national level will be estimated from program perspective. Cost will be estimated for all parties involved with the SSK project implementation namely, service delivery cost for health facilities, overall monitoring and supervision cost for HEU, and scheme management cost for insurance company. To estimate cost, all inputs to be used in SSK project will be identified, quantified and valued. The project and the hospital management personnel will be interviewed for collecting these cost related information. Semi-structured questionnaires will be used for this interview. The inputs will be separated by capital (e.g. Buildings) and recurrent costs (e.g. staff salary). The capital costs will be annualized using their lifetime and 3% discount rate [9, 10]. Total project cost will be estimated by summing up the capital and recurrent costs. The nationwide scale-up cost of the SSK project will be estimated by applying economic modeling and projections technique. The economic modeling of cost will be performed considering the existing utilization of services and unit cost of producing such services. For nation-wide implementation, a hypothetical scenario for cost input (e.g. number of healthcare facilities, additional manpower required) will be prepared in consultation with the experts (e.g. HEU, DGHS personnel and insurance providers). The unit cost information collected from the health facility will be used to estimate cost for this scenario using OneHealth Tool software. A sensitivity analysis of nationwide scale-up cost will be performed considering 5 to 10% increase in utilization of services to realize the situation during full implementation of the project.

### Sample size

#### Quantitative

We use the following formula for estimating sample size to validate the selection process of BPL population and assess knowledge level of SSK card holders, $\mathrm{Sample}\ \mathrm{size}\ \left(\mathrm{n}\right)=\frac{{\mathrm{Z}}_{\left(1-\upalpha \right)/2}^2\times \mathrm{P}\times \left(1-\mathrm{P}\right)}{{\mathrm{L}}^2}$

Where,

n = required sample size,

P = anticipated proportion (positive predictive value/BPL card holders are knowledgeable about the benefit package).

α = size of the critical region (1 – α is the confidence level),

Z_(1-α)/2_ = standard normal deviate corresponding to the specified size of the critical region (α),

L = absolute precision desired on either side (half-width of the confidence interval) of positive predictive value.

We used 95% confidence interval, 5% error level, and 10% non-response for estimating the sample size. Therefore, for validating the selection process of BPL population, an estimated 270 SSK card holders and an equal number of non-card holders will be required to interview assuming positive predictive value at 80%. In total, 540 households (card holders and non card holders) will be interviewed. Similarly, to assess knowledge level of SSK card holders about benefit package, a minimum of 423 BPL card holders will be required to interview assuming 50% of them are knowledgeable.

#### Qualitative

The key-informants will be selected from different level of the project implementation, e.g. the SSK Cell members, scheme operators and service providers. Semi-structured guidelines will be developed based on informants’ characteristics [see Additional file 1]. In phase II and IV of the study, 7 to 9 key-informants will be interviewed. However, actual number will be determined based on data saturation and availability of informants.

### Data analysis

#### Quantitative

Both descriptive and advance analysis will be performed using quantitative data. The positive predictive value will be estimated for validation of BPL population. A 2 × 2 table will be constructed for the poor and non-poor households and the SSK members and non-members households by comparing the poverty line with the household consumption expenditure data. From the table, the probability that a ‘poor’ among those with the BPL population are enrolled in the SSK project (positive predictive value) will be estimated [11].

Factor analysis will be used for ranking the knowledge level of the card holders. Earlier studies have used this technique for assessment of knowledge level [12, 13]. Principle component analysis will be performed to generate the factor score. We will estimate one main factor (namely, knowledge level for SSK benefit package) with items loading on this factor [14]. Using the factor score we will rank household from low to high level of knowledge. Multivariate regression model will be used to assess the association of demographic and socioeconomic characteristics of the respondent with their level of knowledge. In this analysis, level of knowledge will be the dependent variable and age, sex, education level and monthly income will be the explanatory variables.

To understand the service utilization pattern, trend analysis will be performed using project record. Average number of outpatient and inpatient services utilized per 1000 card holders will be estimated for three time points (Table 2). This utilization information will be presented by patient characteristics available in the project record (e.g. age, sex) and cause of illness. This analysis will provide evolving nature of healthcare utilization among the SSK card holders.

Economic modeling and projections will be performed for nationwide cost estimation. Cost per service delivery and cost per beneficiary of SSK project will be estimated considering cost of all parties involved in the project. OneHealth Tool software will be used for nationwide implementation cost estimation.

#### Qualitative

After completion of a KII, a verbatim transcription and translation will be performed immediately using the audiotapes and interview notes. A systematic framework approach will be employed for systematic generation of themes and codes and for analyzing the qualitative data. The Framework Method support thematic analysis in a systematic manner for organization and mapping the qualitative interview data which is appropriate for interdisciplinary and collaborative scheme projects [15]. The research team will become familiar with the whole interview by repeatedly listening the audio recording or by reading the transcript for interpretation. After familiarization with the interview, the researcher will apply ‘code’ that illustrates the interpreted information from the interview for systematic comparison with other components of the dataset. By using the categories and codes, the analytical framework will be applied by indexing subsequent transcripts. For the analysis process, a framework matrix will be generated using spreadsheet and data will be summarized and charting into the matrix by category. Charting ensures data summarization and careful explanation of participant’s own opinion and expressions prior to interpretation by the research team. The interpreted findings under each main theme or category will be presented for the identification of key implementation barriers and possible solution to overcome such barriers. Triangulation of information will be done for findings from different sources.

### Ethical assurance for protection of human rights

This study will involve human subjects hence ethical approval have been obtained from the Research Review Committee and Ethical Review Committee of icddr,b. All respondents of the study will be interviewed after giving written informed consent. Their participation will be voluntary. Efforts will be made to ensure that they are properly informed about the study objectives and thoroughly understand what their participation in the study involves. All collected information will be kept confidential and will be used only for research purposes.

## Discussion

Many people in Bangladesh fall into poverty due to OOP payments for healthcare [2–4]. The introduction of the SSK project in the study Upazila of Bangladesh aims to increase essential services utilization and stimulate better quality of the services through reducing financial burden. This article contains a comprehensive study protocol with the objectives to validate the selection of enrolled BPL population, their knowledge about the scheme, service utilization pattern among them, barriers in service utilization, implementation-related challenges, and cost for scaling up the scheme. This study will provide a comprehensive understanding about the existing challenges of the SSK project to its successful implementation. Through this study, ongoing timely feedback will be provided to the SSK implementer and policymakers in order to have refinement in the implementation strategy.

The rigorous design of the study protocol to capture implementation related challenges of the project is one of the important strengths. This study will collect real-time qualitative and quantitative data over a period of 1 year. The prolonged involvement of the study team will facilitate them to be close to the real implementation scenarios and identify the challenges towards the implementation. The research team will closely collaborate with the key decision makers from the SSK Cell and relevant stakeholders to ensure that the research questions are relevant to the implementation of the project and the evidence generated through the study will be useful in their decision making. This collaboration will not influence the independence of research.

We will start the study activities and share the plan through organizing workshop with the presence of key personnel from the HEU, MoHFW and other relevant organizations. We will share the study findings through reports, policy briefs, and meetings with the local stakeholders. We will also share the learning in the international conferences and publish research papers in the international journals.

One important concern is that, the present study includes the perceptions and strategies of the key stakeholders, implementers and decision makers in the objectives. This may induce biases in their responses. We will be cautious of such possibilities while conducting their interview. We will verify the study findings through comparing information from multiple sources and using different methods of data collection. Another limitation of this study is that the process documentation will be conducted only in the scheme implementation site, which may limit the generalizability of the findings to other regions.

The evidence generated from the study will be useful to program managers for planning nation-wide scale-up accordingly or to replicate such health insurance scheme in similar low-income country settings. The findings will be useful to address financing challenges of healthcare in Bangladesh and for implementation of the healthcare financing strategy developed by the MoHFW of Bangladesh [6]. Methodological challenges of implementation research on health financing schemes would be useful for research communities.

Ultimately, the scientific evidence generated will be used to ensure healthcare for vulnerable groups and subsequently useful for achieving universal health coverage in low- and middle-income countries, which is a global agenda.

## Additional file

Additional file 1: Survey questionnaires and interview guides. The supplementary file consists two appendixes. APPENDINX-A consists quantitative questionnaire for validation study and community survey. APPENDIX-B qualitative interview guides for Key-informant Interviews SSK service providers, insurance scheme management and Health Economics Unit personnel. (PDF 195 kb)

## Abbreviations

BBS: Bangladesh Bureau of Statistics
BPL: Below-Poverty-Line
CBN: Cost-of-basic needs
DGHS: Director General of Health Services
DH: District Hospital
DRG: Diagnosis Related Groups
FGD: Focus Group Discussions
GFA: Gospel for Asia
HCFS: Health Care Financing Strategy
HEU: Health Economics Unit
icddr,b: International Centre for Diarrhoeal Disease Research
KIIs: Key-Informant Interviews
MIS: Management Information system
MoHFW: Ministry of Health and Family Welfare
OOP: Out-of-pocket
SO: Scheme Operator
SSK: Shasthyo Surokhsha Karmasuchi
UpHC: Upazila Health Complex
