# Supplementary material for: Evaluating the implementation related challenges of Shasthyo Suroksha Karmasuchi (health protection scheme) of the government of Bangladesh: a study protocol
Source: BMC Health Serv Res. 2018 Jul 16;18:552. doi: 10.1186/s12913-018-3337-x (PMC6048757; doi:10.1186/s12913-018-3337-x)
Supplement: Supplementary file 1 — Survey questionnaires and interview guides. The supplementary file consists two appendixes. APPENDINX-A consists quantitative questionnaire for validation study and community survey. APPENDIX-B qualitative interview guides for Key-informant Interviews SSK service providers, insurance scheme management and Health Economics Unit personnel. (PDF 195 kb) [file 12913_2018_3337_MOESM1_ESM.pdf]

Supplementary file. Survey questionnaires and interview guides

Appendix A. Validation study and community survey questionnaire

Study title: A study to systematically document the implementation related challenges of *Shasthyo Suroksha Karmasuchi* Health Protection Scheme of the Government of Bangladesh

Principal Investigator: Sayem Ahmed
Organization: International Centre for Diarrhoeal Disease Research, Bangladesh (icddr,b)

Purpose of this research
Greetings! I am Name of the interviewer ---------------- from iccdr,b, an international health research organization. To identify the implementation related challenges of *Shasthyo Suroksha Karmasuchi* health protection scheme of the government, we are conducting a research in this area**.**The purpose of this research is to assess whether the beneficiary under this scheme are identified according to the predefined criterion and provide feedback to the program implementer indentifying the implementation related challenges of the scheme. For this purpose we will ask you about your knowledge of the scheme, your household income, asset, healthcare utilization and related expenses.

Why did we select you?
Since this project is being conducted in this area and you are randomly selected for this study, we have considered you as a respondent of this study.

Method
If you agree to participate in this research study, it would involve an interview lasting about 30 minutes. In this interview, I shall ask you about your income and your time involvement and expenditure to receive this service. You can choose your comfortable place for interview. If you agree I can start interview now or I can come again at your convenient time.

Privacy, anonymity and confidentiality
We are assuring you that information given by you will be kept strictly confidential. We also want to assure you that all records of the interviews will be kept in a safe and secure place for five years and will not be used for any other purposes than the study. We ensure you that your name and other identity will not be exposed while the research results will be published. So it will not be possible to trace the answers back to you.

Future use of information:
Information provided by you will be used for this research only and your name will not be exposed when the research results will be published.

Risk
There are no physical and social risks related to your participation in this study and if you refuse to participate in this study, you and your family will not face any risk.

Benefits
You will not be directly benefited by participating in this study. However**,** this information will help to improve the current project, efficiency and services.

***Freedom of not to participate and withdraw*:**
You are absolutely free to either participate or not participate in the study. You are free not to answer any question if you wish. Even you are free to withdraw at any point of the interview.

Compensation
The study is unable to provide any financial compensation to you.

If you have any queries regarding the study, you are free to ask the interviewer. You can also contact the principal investigator of this study or IRB coordinator at the address given below.

Sayem Ahmed
Principle investigator
icddr,b
Mohakhali, Dhaka-1212
Phone: 02-8860523-32 Ext.2524 or +8801912522643
Email: sayemahmed@icddrb.org

M.A Salam Khan
IRB co-ordinator
Research Administration
icddr,b
Mohakahali, Dhaka-1212
Phone: 02-8860523-32 Ext.3206
Email: salamk@icddrb.org

Are you agree to participate in this study ☐Yes☐No

Signature or left thumb impression of the participant _______________________ Date________________

Signature of the supervisor of the survey _______________________ Date________________

Signature of the Interviewer ______________________ Date________________

Section 1: Identification
**Questionnaire code:**
**Name of the respondent** ____<br>Is the respondent a household head? ......1. Yes ......2. No<br>Address of the respondents-For SSK members write as mentioned in the card**:**<br>____<br>____<br>Union: |___|___| Code-1<br>Mobile number**:** |___|___|___|___|___|___|___|___|___|___|___| **Phone number: Your (1) Not your (2) If others, Please mention the relationship with the person** ____________________________
Is the household under SSK services? ☐1. Yes ☐2. No
Code of the interviewer: |___|___| Interview starting time: |___|___|: |___|___| ☐ am/ ☐ pm<br>Interview end time: |___|___|: |___|___| ☐ am/ ☐ pm<br>Date of the interview:|___|___|:|___|___|:|___|___|___|___|Day/Months/Year<br>Record GPS location:

Code-1 (Union)

1.Kokodhora	8.Narandia
2.Gohaliabari	9.Paikora
3.Doshkia	10.Parokhi
4.Durgapur	11.Bolla
5.Nagbari	12. Bangra
6.Birbasinda	13.Solla
7.Sohodebpur	14.Alenga municipality

Section 2:Information on household members

C1	C2	C3	C4	C5	C6	C7	C8	C9	C10	C11
Household member code		Relationship with the household head<br>1 Self<br>2 Husband/wife<br>3 Father/Mother<br>4 Son/daughter<br>5 Father-in-law / mother-in-law<br>6 Brother/Sister<br>7 Son-in-law / daughter-in-law<br>8 Grandson/Grand- daughter<br>9 Relatives<br>10 Adopted children<br>11 Uncle/aunt<br>12 Grandfather/grandmother<br>13 Relation with who are not relatives<br>14 Others Specify	Gender<br>1 Male<br>2 Female	Age-in integars	Marital status<br>1 Married<br>2 Single<br>3 Divoreced/divorcee<br>4 Separated<br>5 Destitute<br>6 Others- Please specify	Educational qualification of the member in years If doesn't have education write "0"	Main occupation<br>1 Agriculture<br>2 Housewife<br>3 Daylaborer<br>4 Rishkaw/Van driver<br>5 CNG/Auto driver<br>6 Maid<br>7 Fromal Job<br>8 Shopkeeper<br>9 Begger<br>10 Unemployed<br>11 Student<br>12 old<br>13 Child<br>14 Farming<br>15 Sharecropping<br>16 Worker<br>17 Fisherman/oarsman/Fish farming<br>18 Handycraft<br>19 Garments worker<br>20 Reastauratn/worker<br>21 Mechanics<br>22 Salesman<br>23 Barber<br>24 Tailor<br>25 Teacher<br>26 Mobile business<br>27 Not applicable<br>28 Disabled<br>29 Others-specify	Does this member earn regularly? Yes 1 No 2 Not-appilcable 99]	How many days the memebr worked in the last 30 days	Earnings of the last 30 days [In taka]

Section 3: Household information

Part 3A: Housing, land ownership and living standard related information

1	Are you the owner of the homesteadyou used to live?	1. Yes → go question no 3	2. No
2	Who is the owner of the homestead?	1. Others land<br>2. Roadside/govt. land<br>3. Rented<br>(Please mention the area if it is rented)-----sq.ft	
3	How was the living condition of the household? (According to the interviwer)	1. Clean<br>2. Average<br>3. Dirty<br>4. Very dirty	
4	Do you have any other land except the homestead?	1.Yes	2. No
5	Write the amount of land/homestead area	Homestead with pond and pool area:<br>Paki:_______Decimal:_______ = Decimal|___|___|___|___|___| Agricultural land<br>Paki:_______ Decimal: _______ = Decimal|___|___|___|___|___|	
6	How many rooms are there in this household?	Number |___|	
7	What kind of materials was used to make the main room of the household to be verified?	1. Pucca / semi-Pucca<br>2. Tin<br>3. Tin and bamboo<br>4. Tin and others<br>5. Bamboo and others<br>6. Soil<br>7. wood<br>8. ceramics/ tiles<br>9. Others	

	Roof	Wall	Floor
Serial no			

8 Are these goods / assets available at your household? (More than one answer is acceptable?)

No.	goods / assets	Yes	No
1.	Cow/goat	1	2
2.	Fishing net	1	2
3.	duck/Chicken	1	2
4.	Grocery	1	2
5.	Riksha/Van/ Auto Riksha	1	2
6.	Agticultural instruments	1	2
7.	Boat	1	2
8.	Bed	1	2
9.	blanket	1	2
10.	Quilt	1	2
11.	Hurricane	1	2
12.	Chair/ table	1	2
13.	Dining table	1	2
14.	Almirah / Showcase	1	2
15.	Sofa set/ coach	1	2
16.	Television	1	2
17.	Radio/ Tape recorder	1	2
18.	Clock/wall clock	1	2
19.	Telephone/ Mobile Phone	1	2
20.	Bicycle	1	2
21.	Motorcycle	1	2
22.	Freeze	1	2
23.	Fan	1	2
24.	Sewing-machine	1	2
25.	Others specify___________________	1	2

9 Which type of toilet do you use?

No.	Toilet type	Yes	No
1	Pit toilet	1	2
2	Paka toilet	1	2
3	Soil made toilet	1	2
4	Sanitary	1	2
5	Others Specify]______________________	1	2

10 What is the main source of drinking water?

No.	Source type		
1	Tube well	1	2
2	Ponds/rivers/puddles/streams	1	2
3	Rainwater	1	2
4	Pipe/tap/Supply water	1	2
5	Pure/filteredwater	1	2
6	Others please specify_____________________	1	2

11 Do you have electricity connection? 1. Yes 2. No

Part 3B: Household income				
12	Do the household have any permanent income source?	1. Yes	2. No	
14	Is any child of the household working?	1.Yes	2. No	3. Not appilicable
15	In the last 12 months, what were the other sources of household irregular income? Exclude the items considered in the section-2	**No.**	Sources of income	Amount of income in Taka
		1.	Food for work	
		2.	Income from assets	
		3.	VGD/destitute allowance	
		4.	Old allowance	
		5.	Pension	
		6.	Freedom fighter allowance	
		7.	Rremittence	
		8.	Stipend/scholarship	
		9.	Other allowances	
		10.	Others sources specify_____ ________	

Part 3C: Microcredit and loan related information

16	In the last 12 months, was any household member borrow money from any institution?	1. Yes	2. No	
If yes please fill-up the following section				
Member code	What is the source of loan?<br>1. Community scheme<br>2. Small lender organization<br>3. From Bank<br>4. Other Specify].......	How much money did you loan in the last time?	What is the left-over ammount of your loan?	Where did you invest the borrowed money during the last year? Code: 2

Code: 2

1. Poultry farming	10.To buy fishing equipment
2.Cow/goat farming	11.Buy boat
3.Handy crafting	12. Buy van /rickshaw
4.Agriculture	13.Small business
5.House building/repairing	14.Business
6.Marriage	15.Buy agricultural equipment
7.To buy consumption goods	16.Pay the borrowed amount
8.For educational expenses	17.Sent abroad
9.For treatment	18.Others

Part 3D: Food security related information		
18	How often did you eat three `square meals' full stomach meals a day in the past 12 months not a festival day?	1. Mostly 3 meals each day<br>2. Sometimes 3 meals per day<br>3. Rarely 3 meals per day 1-6 times this year<br>4. Never
19	In the last 12 months how often did you yourself skip entire meals because there was not enough food?	1. Never<br>2. Rarely 1-6 times this year<br>3. Sometimes 7-12 times this year<br>4. Often few times each month
20	In the last 12 months how often did you personally eat less food in a meal because there was not enough food?	1. Never<br>2. Rarely 1-6 times this year<br>3. Sometimes 7-12 times this year<br>4. Often few times each month
21	In the last 12 months, how often did you or any of your family have to eat wheat or another grain although you wanted to eat rice not including when you were sick?	1. Never<br>2. Rarely 1-6 times this year<br>3. Sometimes 7-12 times this year<br>4. Often few times each month
22	In the past 12 months how often did your family have to ask food from relatives or neighbors to make a meal?	1. Never<br>2. Rarely 1-6 times this year<br>3. Sometimes 7-12 times this year<br>4. Often few times each month
23	How many months you can live on from your land income?	______Month

Section 4: Household expenditure related information

C1	C2	C3
Sl. no	Types of food	Amount consumed (If does not consumed put “0”)
**Part A: Weekly food expenditure**		
**4.1 Rice consumed in the last week**		
1	Rice coarse (Kg)	
2	Rice-plain (Kg)	
3	Wheat (Kg)	
4	Flour (Kg)	
5	Flattened/Puffed rice(Kg)	
**4.2 Pulse consumed in the last week**		
1	Lentil (kg)	
2	Bengal gram pulses (Kg)	
3	Mush Kalai (Kg)	
4	Pea gram (Kg)	
5	Chick ling-Vetch (Kg)	
6	Green gram (Kg)	
7	Anchor (Kg)	
**4.3 Cooking oil consumed in the last week**		
1	Soybean oil(liter)	
2	Mustard oil (liter)	
3	Dalda (liter)	
4	Ghee (Kg)	
5	Palm Oil (litter)	
6	Others Specify________	
**4.4 Vegetables consumed in the last week**		
1	Amaranth (Kg/bundle)	

2	Malabar spinach (Kg/bundle)	
3	Spinach(Kg/bundle)	
4	Spinach ipomoea aquatic (Kg/bundle)	
5	Others leafy (Kg/bundle)	
6	Others leafy (Kg/bundle)	
7	Others leafy (Kg/bundle)	
8	Potato (Kg)	
9	Pointed gourd(Kg)	
10	Bitter melon(Kg)	
11	Cauliflower (Kg/piece)	
12	Cabbage (Kg/Piece)	
13	Brinja(Kg)	
14	Arum(Kg)	
15	Gourd(Number)	
16	Tomato (Kg)	
17	Green papaya(Kg)	
18	Green banana(Number)	
19	Green chili (Kg)	
20	Onion (Kg)	
21	Garlic (kg)	
22	Ginger (kg)	
23	______Others vegetables(Kg)	
24	_____Others vegetables(Kg)	
25	______Others vegetables(Kg)	
**Part B: Monthly food consumption**		
**4.5** Spices consumption in the last month		
Sl. no	Types of food	Amount consumed (If do not consumed put "0")
C1	C2	C3
1	Dried chili (gm)	
2	Turmeric (gm)	
3	Cumin (gm)	
4	Cinnamon (gm)	
5	Cardamom (gm)	
6	Others spice______(gm)	
7	Others spice_______ (gm)	
8	Others spice_______ (gm)	
**4.6 Fish, meat, egg and milk consumption in the last month**		
1	Large fish (kg)	
2	Small fish kg]	
3	Dried fish (kg)	
4	Beef (kg)	
5	Buffalo meat (Kg)	
6	Local chicken meat (kg)	
7	Poultry chicken meat (Kg)	
8	Duck meat (Kg)	
9	Mutton (Kg)	
10	Egg (Number)	
11	Milk (Litter)	
12	Other protein items__ __(Kg)	
13	Other protein items__ __(Kg)	
14	Other protein items__ __ (Kg)	
**4.7 Fruits consumption in the last month**		
1	Banana (Number)	
2	Chinese (date	
3	Olive (Kg)	
4	Papyaa (Kg)	
5	Mango (Kg)	
6	Jackfruit (Number)	
7	Java Plum (Kg)	
8	Apple (Kg)	
9	Orange (Number)	
10	Graps (Kg)	
11	Guava (Kg)	
12	Pineaple (Number)	
13	Other fruits _________ Kg	
14	Other fruits _________ Kg	
15	Other fruits _________ Kg	
**4.8 Other foods in the last month**		
1	Sugar (Kg)	
2	Jaggery (Kg)	
3	MIlk powder (Kg)	
4	Salt (Kg)	
5	Noodles (Packate)	
6	Semolina (Kg)	
7	Cold drinks (litter)	
8	Sweetned food	

9	__________________ Child food (Kg)	
10	Tea (Kg)	
11	Biscut (Pack)	
12	Bombay mix (Packet)	
13	Others_________ Number	
14	Others_________ Number	
15	Others_________ Number	
**Part C: Tobacco products (weekly expense)**		
**4.9** Tobacco intact in last week		
1	Batel leaf (number)	
2	Betel nut (number)	
3	Tobacco leaf (Packet)	
4	Tobacco (number)	
5	*Gul* (Packet)	
6	*Biri* (Packet)	
7	Cigarette (Packet)	
8	Others_________________Number	
9	Others_________________Number	
10	Others_________________Number	

**Part D: Non-food monthly expenditure**		
C1	C2	C3
**Sl. no**	**Types**	**Expenditure ammount Monthly**
**4.10** Non food expenditure in last month		
1	House rent	
2	Electricity Bill/ solar power	
3	Gas bill	
4	Fuel wood	
5	Fuel oil (kerosene, petrol)	
6	Transport	
7	Health	
8	Education school fees, (coaching, private teacher, etc.)	
9	Notebooks/Pens/books/other study materials]	
10	Communication expense (mobile, telephone, etc.)	
11	Cable TV network bill/ internet bill	
12	Cosmetics expense (Face-powder, facial cream etc.)	
13	Hair Oil/Coconut Oil	
14	Cleaning and hygiene (soap, detergent,hair shampoo etc.)	
15	Laundry	
16	Co-operative instalment/ Micro-credit instalment	
17	Salon/Spa	
18	Others Specify	

**Part E: Non-food yearly expenditure**		
**4.11** Non-food expenditure in last year		
**Sl.no**	**Types**	**Expenditure amount in the last year**
1	Clothing	
2	Shoe	
3	Quilt/quilt/pillow/blanket/bed	
4	Entertainment/religious festival	
5	House construction/House renovation	
6	Furniture	
7	Insurance premium	
8	House keeper/ maids salary	
9	Gifts provide	
10	Donation	
11	Travel	
12	Others Specify	

Section 5: Treatment and expenditure related information of the household members in the last three months (write individual information in separate row)

Does any household member got sick or have symptoms of sickness in the last three months? 1. Yes 2. No

Column 5 – If you didn't seek treatment, why?
1. Problem was not severe
2. The cost for treatment was very high
3. Didn't have enough money
4. Long distance of the health facility
5. No one was available to take to the facility
6. Didn't know the location of facility
7. [If others, specify]

Column 6 – Where did you seek treatment from?
1. Medical College Hospital
2. Specialized hospital
3. [Referral district hospital]
4. Other District Hospital
5. Upazila Health Complex
6. Union Sub center/CC/Rural dispensary
7. Maternal and Child Welfare Center
8. Private Clinic and Hospital
9. NGO Clinic/ Hospital
10. NGO Health Worker
11. Government Health Worker
12. Qualified Private Practitionar
13. Unqalified Private Practitionar
14. Ayurvedic/homeopathic
15. Self-treatment
16. Pharmacy
17. OthersSpecify...]

Column 14 – How did you manage the treatment cost?
1. Regular income
2. Family savings
3. SSK provided
4. Insurance
5. Selling of own materials
6. Selling of Trees/Agricultural crops/domestic animals
7. Selling of permanent property
8. Mortgage of land or property
9. Borrowing money from lender
10. Help from friends or relatives
11. Borrow from Relatives/friends/colleagues
12. Loan from institute/association
13. Others, Please specify

1	2	3	4	5	6		7		8	9										10	11	12	13	14					
Household member code (Section 2)	The disease code of the member	How many days you/ the member suffered from illness?	Did you or the member sought treatment for that disease? Code: Yes1 No2 If answer is no Only question 5 is applicable	If you didn't seek treatment, why?	Where did you seek treatment from?		Did you get admitted to the hospital for that problem? Code: Yes1 No 2; If no then go the question no 9	If yes, how many days you were admitted to the hospital? in full days]	Was your treatment covered by the SSK?	Cost for treatment (If any item is not applicable, palce "0")										How many days did you absent from your work due to illness? Full days	[In the last three months, despite of illness how many days were you present at your work? write "99" if not applicable]	In the last three months, did you left any work permanently due to illness? yes1, No 2, Not applicable 3	In the last 12 months, did any of the household member had to leave school permanently? Yes 1 No 2 Not applicable 3	How did you manage the treatment cost?					
					Sl. no	Source of treatment				Consultaiton fee	Medicine	Bed rent	Diagnostic	Transport	Tips	Attednant	Package	Operation	Others					Source	Amount	Source	Amount	Source	Amount
					1.																								
					2.																								
					3.																								
					1.																								
					2.																								
					3.																								
					2.																								
					3.																								

* 1. Cough2. Fever 3. Malaria 4. Typhoid 5. Diarrhoea/dysentery 6. Vomiting 7.Asthma/breathlessness 8.Minor injury 9. Bone fracture 10. Jaundice 11. Weakness12. Worms 13. Pain/discomfort 14. Skin disease 15. Convulsion/Seizure 16. Dyspepsia 17. Hypertension 18. Diabetes 19. Tuberculosis 20. Leprosy 21. Injury/Paralysis 22. Arthritis 23. Rheumatic Fever 24.Cancer 25. Delivery 26. Obstetric emergency 27.Mental problem 28.Others

Section 6: Effect of illness on food security<br>(If no member of the household suffers from any illness, start from the next section)		
**Sl.no**	**Question**	**Type of Answer**
1	In the last three months, did your family have to cut/reduce consumption expenditure due to the illness of any member?	□ 1. Yes<br>□ 2. No<br>□ 99. Not applicable
2	In the last three months, did your family have to cut/reduce other expenditure e.g., education, clothing, furniture due to the illness of any member?	□ 1. Yes<br>□ 2. No<br>□ 99. Not applicable

Section 7: Effect of Health expenditure and SSK related Question

Sl.no 1	Question	Type of answer	
1	Do you think that many people faces financial catastrophe while seeking healthcare service?	□ 1. Yes □ 2.No	
2	Do you think, some of your neighbour used to work despite of their illness?	□ 1. Yes □ 2. No	
3	Do you know, different institutions used to bear treatment cost of its members?	□ 1. Yes □ 2. No	
4	Have you ever heard of life insurance?	□ 1. Yes □ 2. No	
5	Have you ever heard of health insurance?	□ 1. Yes □ 2. No	
6	Do you know, there is a health protection scheme in this area for the poor people?	□ 1. Yes □ 2. No	
7	Do you know, how SSK services are provided from the healthcare facility?	□ 1. In kind using membership card<br>□ 2. By cash<br>□ 3. Don't know	
8	Do you know, how many disease are covered under SSK scheme?	□ 1. Yes<br>□ 2._____ No (If yes, ask how many?)	
9	What types of treatment expenses are borne by the SSK?Multiple responses are acceptable	1. Free medicine<br>2. Free diagnostic/test<br>3. Referral facility<br>4. Provide expense for referral to District hospital	
10	Where did you learn about the SSK?	1. From others in your locality<br>2. Representative from SSK<br>3. From familiar person<br>4. From local leader<br>5. From Miking<br>6. From poster<br>7. From billboard<br>8. From Television commercial<br>9. Others, please specify	
**Part 7A: SSK service related information**			
11	Card number of SSK<br>(Fill from the card)	|___|___||___|___||___|___||___|___||___|___||___|___||___|___||___|___|___|	
12	Card recieving date	|___|___|:|___|___|:|___|___|___|___|Day/Month/Year	
13	Write the code of card issued person from **Section 2**	|___|___| Member code	
14	Did you ever use SSK card? If didn't use why?	1. Yes	2. No (go to the next section)
15	How many times did you use the card?	___________ times	

Section 8:Health status related question

(In the below table, Each group has three boxes, put tick marks only in one of the boxes that indicate your physical condition.)

Hosuehold member code: (section 2)											
1	**Walking**<br>1) I have no problem in walking<br>3) I have problem in walking<br>3) Cannot move from bed										
2	**Self-care**<br>1) I have no problem with self-care<br>2) I have problemswashing or dressing myself<br>3) I am unable to wash or dress myself										
3	**Usual activities**<br>1) I have no problem with performing my usual activities<br>2) I have some problems with performing my usual activities<br>3) I am unable to perform my usual activities										
4	**Pain/ Discomfort**<br>1) I have no pain or discomfort<br>2) I have moderate pain or discomfort<br>3) I am extreme pain or discomfort										
5	**Anxiety/ Depression**<br>1) I am not anxious or depressed<br>2) I am moderately anxious or depressed<br>3) I am extremely anxious or depressed										
6 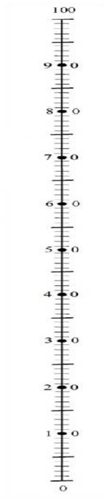 **Indicate today's physical condition in this scale**	To help people say how good or bad a health state is, we have drawn a scale (rather like a thermometer) on which the best state you can imagine is marked 100 and the worst state you can imagine is marked 0. We would like to indicate on this scale how good or bad your own health is today, in your opinion. Please do this by drawing a line from the box below to whichever point on the scale indicates how good or bad your health state is today.<br>Your own health state today										

Thank You

Appendix B. Interview guides for Key-informant Interviews

Interview guide 1. Interview guide to interview SSK service providers

Protocol Number: PR- PR-17047
Protocol Title: A study to systematically document the implementation related challenges of *Shasthyo Suroksha Karmasuchi* (Health Protection Scheme) of the Government of Bangladesh

Principal Investigator: Sayem Ahmed
Organization: International Centre for Diarrhoeal Disease Research, Bangladesh (icddr,b)

1. Identifier
a) Date of interview
b) Name of health facility
c) Name of interviewer

2. Background information
Tell me a little about yourself
Probe: a) Personal background (name, age, education)
b) Current Position (Length of present service, past job experience)
c) Role in the *Shasthyo Suroksha Karmasuchi* (SSK) scheme.

3. What do you think about the *Shasthyo Suroksha Karmasuchi* and the whole process of this scheme? What are the implementation challenges of SSK Scheme?
Probe:

a) What are your responsibilities in SSK scheme?
b) Is there any constraint/barrier that you face while carrying out your duties?
c) What makes it difficult for you during working in the implementation of scheme?
d) Can you manage adequate time to perform your duties?
e) Is there any anticipated challenges for integration of your scheme activities e.g., in identification of BPL population, in card distribution?
f) What kinds of challenges do you face in case of claim management and reimbursements process?
g) What kind on changes or refinements do you think may help you to perform your duties easier?
h) Are you getting support and cooperation from the facilities/management? Who are other people who should be involved in this scheme management process to make the process easier?
i) From your viewpoint, what are the possible barriers to includes the BPL population in t this scheme?
j) What is other people's view about using/adopting the scheme?
k) What are challenges the project may face while nationwide scaling up?

4. How the challenges can be overcome? What are your recommendations in this?
Probe:

a) According to your viewpoint what steps should be taken for addressing the challenges you mentioned?
b) How we could overcome the challenges which integrated with routine activity e.g., BPL identification, card distribution?
c) In your opinion what should be the changes to make smother claim management and reimbursement process?
d) How to ensure/strengthen motivation of related staffs to do the work in a professional manner?

e) How to ensure/strengthen local involvement in this scheme?
f) What is your suggestion to avoid challenges for scaling up of SSK scheme at national level?

Note to interviewer: quickly summarize the major comments heard throughout the interview and ask informants if you covered all the major points. Ask them if there is anything else they would like to tell you that you have not asked them. Finally, thank them for their time.

Interview guide 2. Interview guide for insurance scheme management personnel

Protocol Number: PR- PR-17047
Protocol Title: A study to systematically document the implementation related challenges of *Shasthyo Suroksha Karmasuchi* (Health Protection Scheme) of the Government of Bangladesh
Principal Investigator: Sayem Ahmed
Organization: International Centre for Diarrhoeal Disease Research, Bangladesh (icddr,b)

1. Identifier
a) Date of interview
b) Name of health facility
c) Name of interviewer

2. Background information
Tell me a little about yourself

Probe: a) Personal background (name, age, education)
b) Current Position (Length of present service, past job experience)
c) Role in the *Shasthyo Suroksha Karmasuchi* (SSK) scheme.

3. What do you think about the *Shasthyo Suroksha Karmasuchi* and the whole process of this scheme? What are the implementation challenges of SSK Scheme?
Probe:

a) What are your responsibilities in SSK scheme?
b) Is there any constraint/barrier that you face while carrying out your duties?
c) What make it difficult for you during working in the scheme implementation?
d) Can you manage adequate time to work in the implementation process?
e) Are there any anticipated challenges for BPL identification and card distribution?
f) What kind of challenges do you face in times of providing management support to the hospitals?
g) What kind of challenges do you face in times of SSK patient's entry to hospital?
h) What are the challenges associated with sending claim documents to HEU and reimbursement to the provider?
i) Are you getting support and cooperation from the facilities/management? Who are other people who should be involved in this scheme management process to make the process easier?

4. How the challenges can be overcome? What are your recommendations in this?
Probe:

j) From your viewpoint, what are the possible suggestion that can improve the patients registration and enrollment process?
k) What kind of steps can be taken to improve the claim processing, prompt verification, and reimbursement situation?
l) How to ensure/strengthen motivation of related staffs to do the work in a professional manner?
m) In your opinion, what can be the future action plan to implement this scheme successfully?
n) What are challenges for implementing the scheme in a larger scale?

Note to interviewer: quickly summarize the major comments heard throughout the interview and ask informants if you covered all the major points. Ask them if there is anything else they would like to tell you that you have not asked them. Finally, thank them for their time.

Interview guide 3. Interview guide for Health Economics Unit personnel

Protocol Number: PR- PR-17047
Protocol Title: A study to systematically document the implementation related challenges of *Shasthyo Suroksha Karmasuchi* (Health Protection Scheme) of the Government of Bangladesh
Principal Investigator: Sayem Ahmed
Organization: International Centre for Diarrhoeal Disease Research, Bangladesh (icddr,b)

1. Identifier
a) Date of interview
b) Name of health facility
c) Name of interviewer

2. Background information
Tell me a little about yourself
Probe: a) Personal background (name, age, education)
b) Current Position (Length of present service, past job experience)
c) Role in the Shasthyo Suroksha Karmasuchi (SSK) scheme.

3. What do you think about the *Shasthyo Suroksha Karmasuchi* and the whole process of this scheme? What are the implementation challenges of SSK Scheme?
Probe:

a) What are your responsibilities in SSK scheme?
b) Is there any constraint/barrier that you face while carrying out your duties? How did they make obstacle to your duties?
c) What are the challenges of the existing system for verification of the claim documents claim management and reimbursement?
d) Do you feel you have enough time to perform your job responsibility while doing SSK implementation related activities?
e) What kind of changes may be helpful for performing your duties?
f) Are you getting support and cooperation from the facilities/management? Who are other people who should be involved in this scheme management process to make the process easier?

4. How the challenges can be overcome? What are your recommendations in this?
Probe:

g) From your viewpoint, what are the possible suggestions that can improve the problems that you have just mentioned?
h) What kind of steps can be taken to improve the claim processing, prompt verification, and reimbursement situation?
i) How to ensure/strengthen motivation of related staffs to do the work in a professional manner?
j) In your opinion, what can be the future action plan to implement this scheme successfully?
k) What are challenges for implementing the scheme in a larger scale?

Note to interviewer: quickly summarize the major comments heard throughout the interview and ask informants if you covered all the major points. Ask them if there is anything else they would like to tell you that you have not asked them. Finally, thank them for their time.
